# Supplementary material for: Leukocyte Nuclear Morphology Alterations in Dilated Cardiomyopathy Caused by a Lamin AC Truncating Mutation (LMNA/Ser431*) Are Modified by the Presence of a LAP2 Missense Polymorphism (TMPO/Arg690Cys)
Source: Int J Mol Sci. 2022 Nov 7;23(21):13626. doi: 10.3390/ijms232113626 (PMC9656322; doi:10.3390/ijms232113626)
Supplement: Supplementary file 1 [file ijms-23-13626-s001.zip › ijms-1975710-supplementary.pdf]

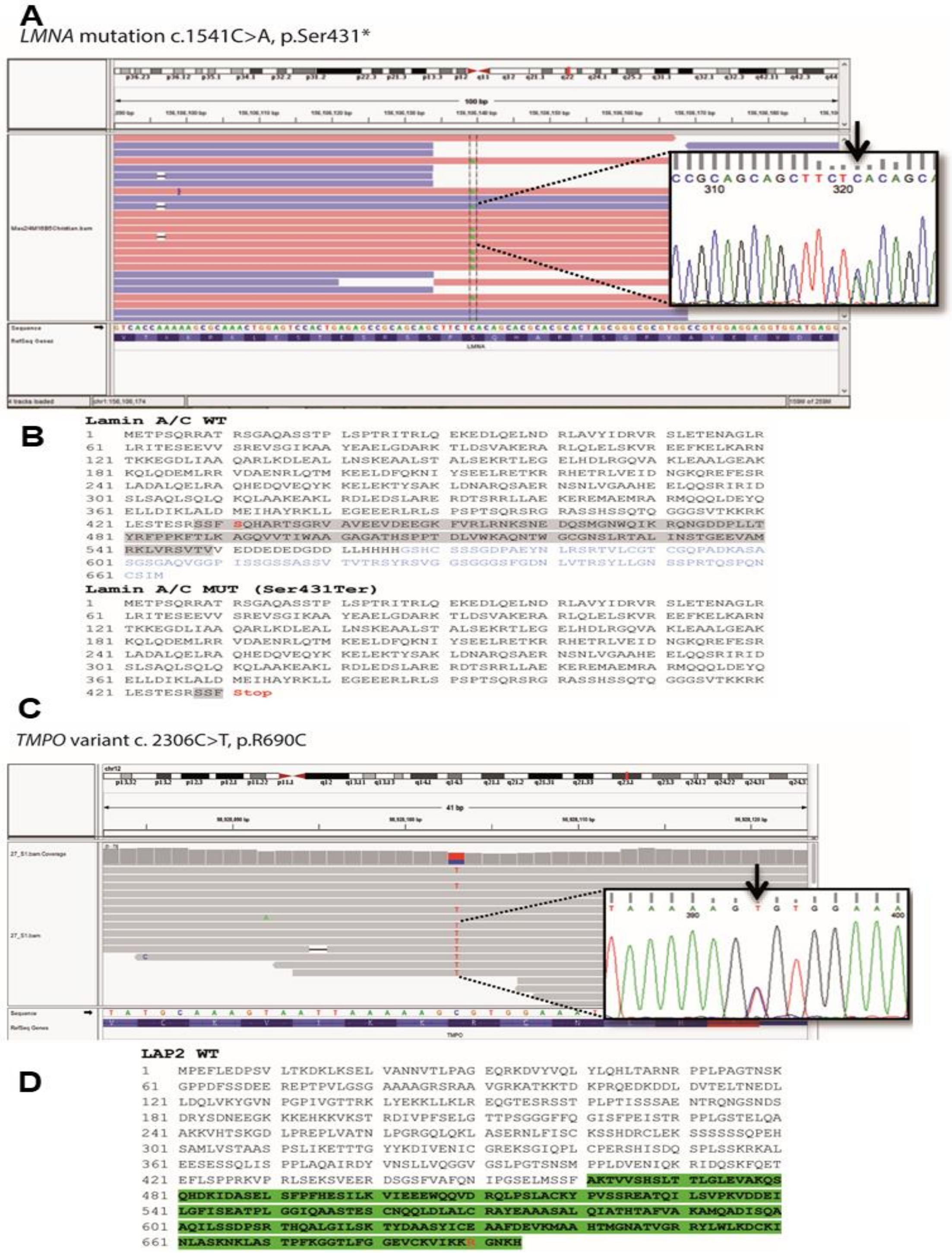

Figure S1. *LMNA* Ser431* and *TMPO* Arg690Cys variants. (**A**) The *LMNA* Ser431* mutation. observed on the Integrative Genomics Viewer with its corresponding electropherogram. (**B**) Full amino acid sequence lamin AC (upper panel). Amino acids forming lamin A are indicated in black and blue, while amino acids forming lamin C are indicated in black. The lower panel shows the sequence of the truncated Ser431* protein. (**C**) The *TMPO* Arg690Cys variant observed on the Integrative Genomics Viewer with its corresponding electropherogram. (**D**) Full amino acid sequence of the LAP2α protein. Arg690 is indicated in red.

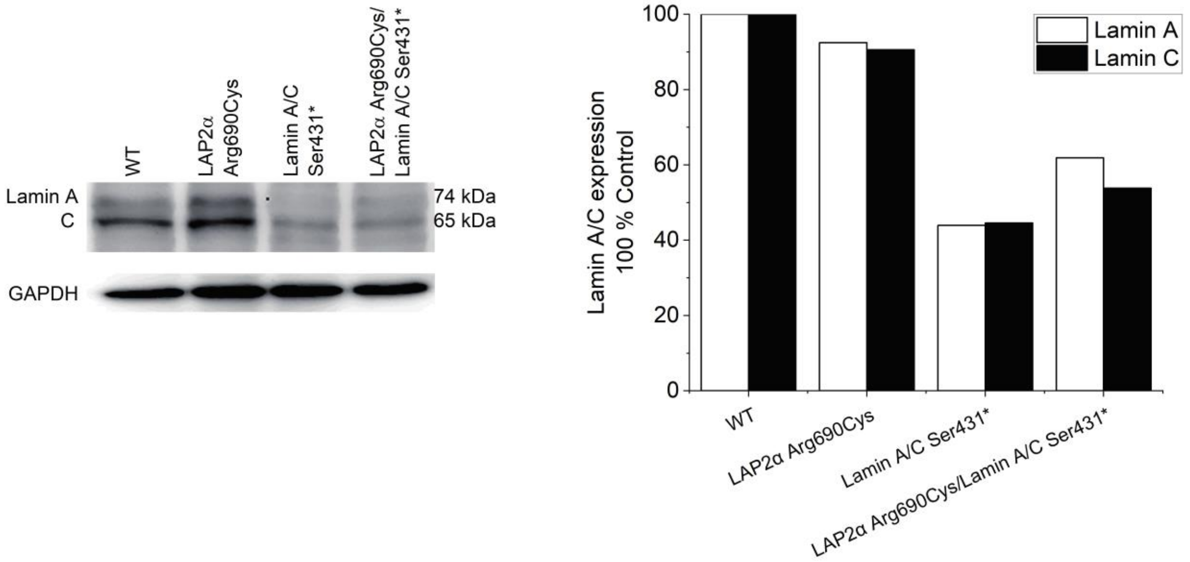

Figure S2. **Lamin A and C protein expression in leukocytes from individuals with different genotypes**. Wildtype (III-5), LAP2α Arg690Cys heterozygous (III-6); lamin AC Ser431* heterozygous (IV-1); and lamin AC Ser431*/ LAP2α Arg690Cys compound heterozygous (IV-2) individuals. Only one experiment was performed per patient, patients were not available for replication experiments.

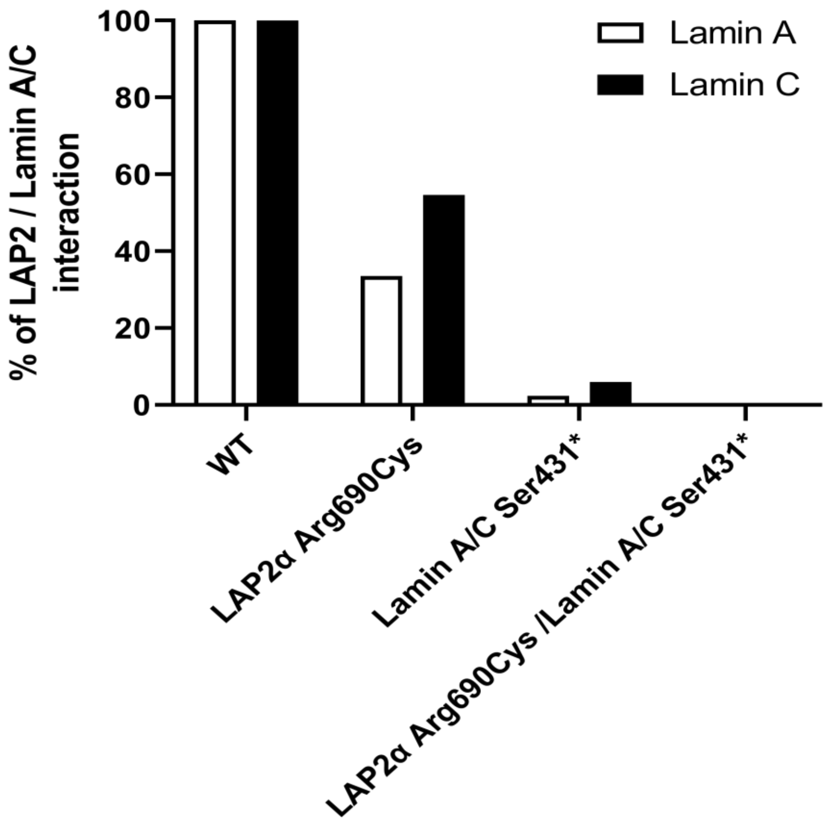

Figure S3. Lamin A/C and LAP2α co-immunoprecipitation analysis. Leukocyte extracts obtained from wildtype (III-5), LAP2α Arg690Cys heterozygous (III-6); lamin AC Ser431* heterozygous (IV-1); and lamin AC Ser431*/ LAP2α Arg690Cys compound heterozygous (IV-2) individuals. Only one experiment was performed per patient, patients were not available for replication experiments.
